# Capsulated *Lactococcus garvieae* caused devastating mortality in Atlantic bluefin tuna, *Thunnus thynnus*: genomic and histopathologic characterization

**DOI:** 10.3389/fcimb.2026.1831351

**Published:** 2026-07-03

**Authors:** Muhammed Duman, Abigail Armwood, Nihed Ajmi, Görkem Taşçı, David Speare, Özkan Yavaş, Izzet Burcin Saticioglu

**Affiliations:** 1 Department of Aquatic Animal Diseases, Faculty of Veterinary Medicine, Bursa Uludag University, Bursa, Türkiye; 2 Department of Population Health and Pathobiology, College of Veterinary Medicine, North Carolina State University, Raleigh, NC, United States; 3 Department of Pathology & Microbiology, Atlantic Veterinary College, University of Prince Edward Island, Charlottetown, PE, Canada; 4 Department of Pathology, Faculty of Veterinary Medicine, Bursa Uludag University, Bursa, Türkiye

**Keywords:** capsulated Lactococcus garvieae, Atlantic bluefin tuna, lactococcosis, climate change, summer outbreak

## Abstract

Atlantic bluefin tuna (ABFT; *Thunnus thynnus*) is among the most valuable commodities in Mediterranean mariculture, and recent increases in seawater temperatures have coincided with the re-emergence of bacterial diseases causing catastrophic losses. During the summer 2025 mortality event, we investigated stranded and moribund ABFT using bacteriological isolation and identification, high-throughput 16S amplicon profiling of tissue-associated bacterial communities, whole-genome sequencing to resolve a complete genome of the etiologic agent, transmission electron microscopy, and gross and histopathological examinations. Across multiple organs, the metagenomic profiles were overwhelmingly dominated by *Lactococcus garvieae*, supporting a primary systemic bacterial etiology. The isolate displayed a capsulated phenotype, and genome analysis identified a capsule-associated gene cluster consistent with a capsulated lineage. Capsule expression was further confirmed ultrastructurally by transmission electron microscopy. Pathology indicated fulminant septicemia with prominent hemorrhagic lesions and severe cardioperitoneal involvement, including fibrinous epicarditis with abundant Gram-positive cocci, alongside marked hepatic and splenic pathology. Collectively, these data document, for the first time in two decades, the detection of a capsulated *L. garvieae* serotype or lineage associated with ABFT mass mortality. Rapid etiologic confirmation, mitigation of temperature-related and husbandry-associated stress, and targeted prevention strategies (including vaccination and biosecurity) are recommended to reduce recurrence in warming coastal waters.

## 1 Introduction

Aquaculture production has expanded rapidly on a global scale, with increases recorded across nearly all farmed fish species, including rainbow trout, sea bream, sea bass, Nile tilapia, carp, and catfish (FEAP, 2025). Concurrent with this growth has been an increase in reports of infections caused by diverse pathogens. Within the Mediterranean basin, Atlantic bluefin tuna (*Thunnus thynnus*, ABFT) is the principal tuna species of commercial interest for both capture fisheries and tuna aquaculture (Ottolenghi, 2008; Balli et al., 2016; FAO, 2024). ABFT is distinguished by its elevated lipid content, a characteristic that results in premium prices within the Japanese market. Its unit value exceeds that of most other commercial fish species, primarily because of the sustained demand from high-end sashimi markets in Japan. The mean reported price for export-grade fish is approximately USD 36,700 per ton (approximately USD 36.7 per kg) (NOAA Fisheries, 2019; The Pew Charitable Trusts, 2020; Maar et al., 2022). Furthermore, production has increased in recent years, from 25,096 tons in 2020 to 25,700 tons in 2021 and 32,200 tons in 2023 (FEAP, 2025). Given an average harvest weight of approximately 200 kilograms, the value per individual can approach USD 8,000, thus placing ABFT among the highest-value finfish globally (Jelić Mrčelić et al., 2023). The primary producers of fattened ABFT are Spain, Croatia, and Malta, with supplementary production from Greece, Italy, Morocco, Portugal, Tunisia, and Turkey (FEAP, 2025).

Over the past two decades, the ABFT aquaculture industry has undergone significant expansion, with no evidence of widespread mortality events among fish populations. Routine harvesting practices have been in place. However, recent reports indicate an increase in acute mortalities during the summer months, coinciding with periods of elevated Mediterranean Sea temperatures ranging from 25 to 30 °C, particularly in offshore farming settings (https://pirireis.mgm.gov.tr/deniz-suyu-sicakliklari). A considerable number of ABFT have been discovered to be deceased or moribund along the coastline. During the summer of 2025, unusual strandings and mortalities of Atlantic bluefin tuna were reported along the İzmir coastline, raising concern regarding an emerging infectious disease event (Gündeme Bakış, 2025). In the present study, we investigated affected Atlantic bluefin tuna submitted for diagnostic work-up to confirm the etiologic agent and characterize the associated pathology and genome. Mortalities were observed both among free-ranging fish stranded along nearshore areas (drawing public attention and prompting assessments by governmental authorities) and within sea-cage farming operations. Although *L. garvieae* is well recognized as a cause of major losses in finfish aquaculture, with outbreaks frequently linked to warm-water conditions (Meyburgh et al., 2017; Shahin et al., 2022; Rao, 2023; Salogni et al., 2024), the recent warming of marine waters has coincided with increased reporting of *L. garvieae*–associated disease in marine aquaculture. The first documented capsulated serotype designated KG– was reported in 2002 (Kawanishi et al., 2006). Morita et al. (2011) showed that the capsulated Lg2 strain (KG–) exhibits greater virulence in yellowtail (*Seriola quinqueradiata*) than the non-capsulated ATCC 49156 strain (KG+) described by Ooyama et al. (2002). Following the initial description of Lg2, however, KG–/capsule-positive isolates supported by genomic evidence (i.e., an identifiable capsule gene cluster) have been reported only rarely and have not been widely confirmed across subsequent *L. garvieae* outbreaks. Instead, several studies have described capsule-like surface structures, including hyaluronic acid–rich cell walls, without demonstrating an Lg2-like capsule locus (Rao et al., 2022). Accordingly, the present isolate represents one of the rare post-Lg2 reports documenting a capsule-associated gene cluster together with phenotypic capsulation. In recent years, the threat posed by *L. garvieae* to aquaculture has intensified; in parallel with rising sea temperatures across the Mediterranean, the earliest rigorously documented outbreaks in gilthead seabream (*Sparus aurata*) from the Gulf of Follonica (Tuscany, Italy) indicate an expanding marine host range and temperature-linked risk (Coltraro et al., 2025).

The present study examined a series of cases involving more than 30 ABFT that became stranded or moribund during the summer of 2025; following submission of the fish to the Animal Hospital, Faculty of Veterinary Medicine, for necropsy, the specimens were transported to our laboratory within 1–3 h for microbiological and pathological examinations. By conducting a range of gross pathologic, histopathologic, and microbiological analyses, we identify the agent responsible for mortality and provide genomic characterization of the capsulated *L. garvieae*. In the literature on *Lactococcus*, capsulation has sometimes been discussed alongside historical serotyping conventions (e.g., KG terminology). In the present study, we use the terms capsulated or capsule-positive strictly to denote phenotypic capsule expression supported by TEM and genomic evidence. This is the first such report since 2002, and it is accompanied by a detailed account of the disease’s pathologic presentation.

## 2 Materials and methods

### 2.1 Fish and necropsy

In late July 2025, at marine water temperatures of 22–25 °C in İzmir Province, ABFT exhibited clinical signs including beaching, unstable swimming near the shoreline, surface persistence, and markedly reduced swimming speed (approximately 5–10 individuals per day). Examinations were conducted in regions with sea-cage ABFT aquaculture and in adjacent nearshore coastal areas, where beached animals were retrieved and assessed. Reports indicated that 1, 500–2, 000 individuals died during the event (Gündeme Bakış, 2025). In the study area, mortalities among caged ABFT reportedly increased toward mid-August, decreased toward the end of August, and continued to decline in September (personal communication).

In this study, moribund fish were presented to the Veterinary Faculty Animal Hospital for clinical examination, necropsy, microbiological analysis and other routine diagnostic tests. All fish included in laboratory-based analyses (n=30) were obtained as moribund and recently dead individuals collected from the coastline, the vicinity of sea-cage farming sites, or both and submitted to our laboratory for diagnostic evaluation. These 30 fish (small, n=10, ~50 kg; large, n=20, ~150 kg) were collected across the early, mid, and late phases of the outbreak and comprised the study set used for detailed laboratory work-up (microbiological, histopathological, molecular, and genomic analyses as described below). Histopathology was performed on selected tissues from the laboratory-submitted study set (n=30), prioritizing cases representing mid-to-late disease. Detailed farm-internal operational records (e.g., cage-level inventories and mortality logs) were not available to the authors and are held by the respective companies and governmental authorities. For diagnostic evaluation, affected ABFT were delivered to our laboratory within 1–3 h under cold-chain conditions for microbiological and pathological examinations.

The external examination included inspection of the eyes, mouth, gills, fins, skin, anus, and overall body integrity. Internally, the liver, spleen, gonads, stomach, intestines, heart, kidneys, and coelomic cavity were systematically evaluated for gross abnormalities. Microbiological cultures were performed aseptically from both healthy-appearing and affected tissues. Following the aseptic removal of the skin, tissue samples from the body wall skeletal muscle, liver, and spleen were collected aseptically for 16S amplicon sequencing analysis. All specimens were screened for ectoparasites using wet mounts of gill filaments, fin clips, and skin and lesion scrapings. Additionally, fish were examined for trauma related to husbandry, capture, or handling as well as gross indicators of nutritional disorders. Representative samples of baitfish used as feed were assessed microbiologically and screened for specific toxins using the institution’s standard diagnostic panel. Representative bait and feed fish were screened for evidence of acute toxicosis using the routine diagnostic panel available at our institution.

### 2.2 Bacterial isolation and identification

Sterile loop or swab samples were collected from the subcutaneous muscle layer, liver, spleen, kidney, fluid-filled abscesses in the spleen, heart, cream-like exudates from the gut and liver, and ocular tissues from all animals (n = 30). Samples were inoculated onto Marine Agar 2216 (BD, 279110), tryptic soy agar (TSA; Merck, 105458), thiosulfate–citrate–bile salts–sucrose agar (TCBS; Merck, 103854), TSA supplemented with 1.5% sea salt (Sigma-Aldrich, S9883), and blood agar prepared from blood agar base (BA, Merck, 110886) supplemented with 5% defibrinated sheep blood. Inoculations were performed in duplicate and incubated at two temperatures, 15 °C and 22 °C. Colonies that grew were subjected to standard preliminary microbiological tests, including Gram staining, oxidase and catalase activity tests, motility tests, and glucose fermentation in oxidation-fermentation (O/F) medium (OF Basal Medium; Merck, 110990). For isolates presumptively consistent with streptococci/enterococci (Gram-positive, catalase-negative cocci), biochemical profiling was additionally performed using the API Rapid ID 32 Strep system (bioMérieux) in accordance with the manufacturer’s instructions. Subsequently, the isolates were examined by matrix-assisted laser desorption/ionization time-of-flight mass spectrometry (MALDI-TOF MS) for primary identification. Species identification was performed by MALDI-TOF MS using a Bruker [smartflex] instrument (Bruker Daltonics, Bremen, Germany) operated with MBT Compass software (v4.1) and the Bruker Biotyper reference library (DB-12438). Colonies were processed using the standard formic acid extraction procedure according to the manufacturer’s instructions, and spectra were acquired in linear positive mode and matched to the database to obtain Biotyper log(score) values. Species-level confirmation was then performed using the primers reported by Saticioglu et al. (2023).

### 2.3 16S amplicon sequencing and analysis

Tissues were collected under aseptic conditions to minimize environmental carryover. After reflecting the skin with sterile forceps and a scalpel, the internal muscle was excised using a new, single-use blade. Additional material was sampled aseptically from splenic and hepatic lesions, with fresh instruments used for each specimen. Approximately 20–50 mg of tissue was transferred to 2 mL screw-cap tubes preloaded with one 5 mm sterile stainless-steel bead and lysis buffer (lysozyme-based enzymatic lysis buffer (Qiagen, cat. no. 56304)). Samples were homogenized on a TissueLyser II (Qiagen, Germany) at 50 Hz for 60 seconds, with brief cooling on ice between cycles to prevent overheating, yielding uniform suspensions suitable for nucleic acid purification.

Genomic DNA was then isolated using a magnetic-bead–based extraction kit (Nucleogene, Türkiye), following the manufacturer’s instructions for tissue inputs. DNA concentration and purity were quantified using NanoDrop spectrophotometry (Multiskan Go, Thermo Fisher Scientific). The bacterial 16S rRNA gene was amplified using the universal primer pair 27F (5′-AGAGTTTGATCMTGGCTCAG-3′) and 1492R (5′-TACGGYTACCTTGTTACGACTT-3′), targeting the hypervariable regions V1–V9 to enable broad taxonomic resolution (Lane, 1991). PCR amplification was performed using Q5 High-Fidelity DNA Polymerase (New England Biolabs), selected for its low error rate to ensure accurate generation of near-full-length 16S amplicons. Sequencing was performed on the Oxford Nanopore PromethION platform using FLO-PRO114M flow cells and the SQK-NBD114.24 library preparation kit. Basecalling was performed with Guppy v6.3.7 (Oxford Nanopore Technologies) using the Super Accuracy (SUP) model. Reads with a minimum Q-score of 15 were retained to ensure high-quality input (Lane, 1991; Knight et al., 2018; Li, 2018; Tyler et al., 2018; Johnson et al., 2019). Raw reads were processed using the epi2me-labs/wf-16s workflow (v1.3.0) provided by Oxford Nanopore Technologies. To reduce artifacts and incomplete sequences, reads were filtered to include only those between 1000 and 1850 bp in length, corresponding to the expected size range of full-length 16S rRNA gene amplicons (Johnson et al., 2019). Taxonomic classification was performed using minimap2 (v2.26-r1175) (Li, 2018) for sequence alignment against the NCBI 16S/18S RefSeq database. An identity threshold of 85% was applied to ensure reliable classification while maintaining broad taxonomic depth. To minimize the influence of sequencing noise and background contaminants, a relative abundance cutoff of 0.01% was used to exclude low-frequency taxa, consistent with current best practices in microbiome profiling (Knight et al., 2018).

### 2.4 Genome sequencing and analysis

The genomic DNA was extracted from the isolates using a commercial kit as described in section 2.3 (Nucleogene, Türkiye). Purity and concentration were measured with a NanoDrop spectrophotometer (Multiskan Go, Thermo Fisher Scientific) and a Qubit 4 fluorometer (Invitrogen, USA). For long-read sequencing, 400 ng of high-molecular-weight genomic DNA was used without fragmentation, following the standard Oxford Nanopore Technologies (ONT) protocol. Library preparation was performed using the Ligation Sequencing Kit (SQK-NBD114-24, Oxford, UK), and sequencing was carried out on a P2 Solo system using a PromethION Flow Cell (R10.4.1) for 24 hours. Short-read sequencing libraries were prepared using the Nextera XT DNA Library Preparation Kit (Illumina, San Diego, CA, USA) and sequenced on the Illumina NovaSeq 6000 platform with a 2 × 150 bp paired-end configuration and a 1000-cycle HiSeq reagent kit. A hybrid *de novo* assembly was generated using Unicycler (v0.4.6) on the Bacterial and Viral Bioinformatics Resource Center (BV-BRC) platform (Wick et al., 2017; Olson et al., 2023). Raw sequence reads were processed using Trim Galore (v0.6.5dev) for adapter removal and quality filtering. Polishing was performed using Racon (v1.5.0) based on long-read mappings and Pilon (v1.24) based on short-read alignments produced with Bowtie2 (v2.4.4) and processed with SAMtools (v1.20). Assembly quality was evaluated with QUAST (v5.2.0) (Gurevich et al., 2013). Digital DNA–DNA hybridization (dDDH) values were determined using the Type Strain Genome Server (TYGS) to assess the taxonomic placement of the isolates in comparison with type strains (Meier and Göker, 2019).

Multilocus sequence typing (MLST) analysis was performed using the *L. garvieae* PubMLST scheme with assembled genome sequences as input, following the MLST framework previously described for *L. garvieae* and the allele-based curation system implemented in PubMLST (Ferrario et al., 2013; Jolley et al., 2018). Sequence type (ST) assignment was based on the seven housekeeping loci *als, atpA, galP, gapC, gyrB, rpoC*, and *tuf*. The corresponding locus sequences were retrieved from each genome assembly and compared against the reference alleles available in the PubMLST database for allele number and ST determination. For comparative phylogenetic analysis, *L. garvieae* isolates deposited in the PubMLST database were included, and concatenated nucleotide sequences of the seven MLST loci were used to generate a sequence-based phylogenetic tree. Tree visualization and annotation were performed using the Interactive Tree of Life (iTOL) v5 (Letunic and Bork, 2021).

To construct an *L. garvieae* comparative genome set, GenBank was queried on 15 September 2025 and 145 genomes were individually inspected. Seventeen records annotated as “unverified organism” and twenty-seven metagenome-assembled genomes (MAGs) were excluded, and three additional entries were removed as duplicate registrations under the same isolate name. The remaining candidates were taxonomically validated using the Type Strain Genome Server (TYGS), resulting in the exclusion of four genomes that did not resolve to *L. garvieae*. The curated dataset was then used for downstream analyses of antimicrobial resistance (AMR) and virulence genes. A total of 98 taxonomically verified *L. garvieae* genomes were retained in the final dataset and used for comparative analyses.

A core-genome phylogeny was reconstructed using the curated set of 98 taxonomically validated *L. garvieae* genomes. To ensure uniform annotation, all genomes were processed with Bakta (Schwengers et al., 2021). Pangenome analysis and filtered core-genome alignment were generated with Panaroo using strict mode and a 95% core-gene threshold (Tonkin-Hill et al., 2020). Maximum-likelihood analysis was performed with IQ-TREE using ModelFinder, 1, 000 SH-aLRT replicates, and 1, 000 ultrafast bootstrap replicates (Kalyaanamoorthy et al., 2017; Minh et al., 2020; Hoang et al., 2018).

Comparative genomic analyses were performed to assess genome structure and capsule locus organization among selected *L. garvieae* strains. Comparisons included the study isolates, the previously reported capsulated reference strain Lg2, the capsule-negative reference strain ATCC 49156, and additional *L. garvieae* plasmid and genome sequences relevant to capsule locus organization, including pNUF18, pNUF462, pNs15sousui29, and pBSLG13015. Both circular whole-genome comparisons and linear plasmid-region comparisons were generated in Proksee based on BLAST alignments, allowing visualization of genome synteny, GC content, GC skew, and conservation of the capsule gene cluster together with its flanking elements (Grant et al., 2023).

The presence of virulence-associated genes was evaluated using *in silico* PCR analysis. A total of 14 virulence genes related to hemolysin (*hly-1*, *hly-2*, *hly-3*), adhesion (*adh*, *adhCI*, *adhCII*, *adhPavA*, *adhPsaA*, *LPxTG-1–4*), enzyme (*eno*, *pgm*, *sod*, *NADH oxidase*), and capsule (*Capsule*) functions were screened. Primer sequences were adopted from previously published studies (Colussi et al., 2023). Analyses were performed using the *primersearch* program implemented in the EMBOSS suite (Rice et al., 2000), and the results were summarized as a binary presence or absence matrix for comparative visualization.

Antimicrobial resistance (AMR) genes were identified using the NCBI AMRFinderPlus tool (Feldgarden et al., 2019), which detects acquired resistance genes, point mutations, and stress response elements based on curated reference databases. Genome assemblies were analyzed with default parameters, and identified AMR determinants were classified according to antimicrobial class and resistance mechanism.

### 2.5 Transmission electron microscopy

Capsule formation was evaluated after growth on Todd–Hewitt medium (Becton Dickinson and Company) as described previously (Ooyama et al., 2002; Altun et al., 2025). Cell size, morphology, and capsule expression were examined by transmission electron microscopy using cells harvested during the exponential growth phase after incubation for 20 h at 28 °C. Samples were negatively stained with 1% phosphotungstic acid (pH 7.0), and imaging was performed with a Hitachi H600 electron microscope at 75 kV (Ooyama et al., 2002; Altun et al., 2025). Because slide agglutination serotyping was not performed, we refrain from assigning the isolates to established serotypes and interpret our findings at the levels of capsule expression and capsule-locus genetics.

### 2.6 Histopathology

For histopathology, tissues were collected from laboratory-submitted fish (n=30 study set), focusing on cases and organs with gross lesions and representing the predominant mid-to-late disease presentation. Immediately after excision, specimens were immersed in 10% neutral buffered formalin at a fixative-to-tissue volume ratio of approximately 10:1 for 12–24 hours, with the duration extended to 48 hours for thicker pieces. Tissue slices were kept at a thickness of ≤5 mm to ensure adequate penetration of the reagents. Tissues were then processed to formalin-fixed, paraffin-embedded (FFPE) blocks and sectioned for hematoxylin–eosin staining in accordance with established histopathological protocols. Select sections were additionally stained with Gram stain to highlight bacterial agents.

### 2.7 Antimicrobial susceptibility

The antimicrobial susceptibility of the disease agent was assessed according to the CLSI guidelines (CLSI, 2014; Miller et al., 2014). Minimum inhibitory concentrations (MICs) were determined by broth microdilution in cation-adjusted Mueller–Hinton broth supplemented with 2.5% lysed horse blood (CAMHB+LHB), with an inoculum of approximately 5 × 10^5^ CFU mL^−1^ per well. MIC testing included antimicrobials commonly used in aquaculture: enrofloxacin, erythromycin, florfenicol, doxycycline, oxytetracycline, and sulfamethoxazole across a concentration range of 0.015 to 250 μg/mL^-1^ at 28 °C for 24h. Disc diffusion testing was performed on Mueller–Hinton agar using trimethoprim–sulfamethoxazole (SXT, 1.25/23.75 μg), erythromycin (E, 15 μg), doxycycline (DO, 30 μg), oxytetracycline (OT, 30 μg), florfenicol (FFC, 30 μg; Oxoid), ciprofloxacin (CIP, 5 μg), and enrofloxacin (ENR, 5 μg); MIC testing and disk diffusion were performed following CLSI methodology (VET03/VET04) at 28 °C 24h. Because CLSI/EUCAST interpretive breakpoints are not available for *L. garvieae*, results were reported descriptively (MIC values and inhibition zones) without categorizing isolates as susceptible, intermediate or resistant; quality control strains were interpreted using CLSI-recommended QC ranges. *Escherichia coli* ATCC 25922 and *Aeromonas salmonicida* subsp. *salmonicida* ATCC 33658 served as quality control strains.

## 3 Results

### 3.1 Gross and histopathologic examination findings

The temporal progression described below is based on combined field observations (on-site necropsies) and laboratory submissions collected across outbreak phases, whereas laboratory-based assays are restricted to the defined n=30 study set. In the first week of August, only Atlantic bluefin tuna (ABFT) swimming near the shoreline or stranded individuals were reported; macroscopic examination of the fish revealed no external lesions. During the same period, observations approximately one nautical mile offshore noted ABFT at the surface exhibiting laterally recumbent swimming or slow swimming. Macroscopic inspection of the fish brought aboard showed no external lesions, although corneal opacity was present in some individuals (**Supplementary Figure 1**). Over the course of the outbreak, a total of 30 representative Atlantic bluefin tuna, including small fish (n = 10, mean weight = 50 kg) and large fish (n = 20, mean weight = 150 kg), were collected and submitted for systematic postmortem evaluation, microbiological analysis, and subsequent molecular and genomic investigations. Wet-mount examinations of gill filaments, fin clips, and skin and skin lesion scrapings did not reveal ectoparasites in the examined fish. Gross evaluation did not identify lesions suggestive of a primary feed-associated disorder beyond the systemic hemorrhagic presentation described below. Microbiological screening of representative bait and feed fish did not yield consistent bacterial findings that could explain the outbreak, and no evidence of acute toxicosis was detected in the targeted toxin screen.

**Figure 1:**
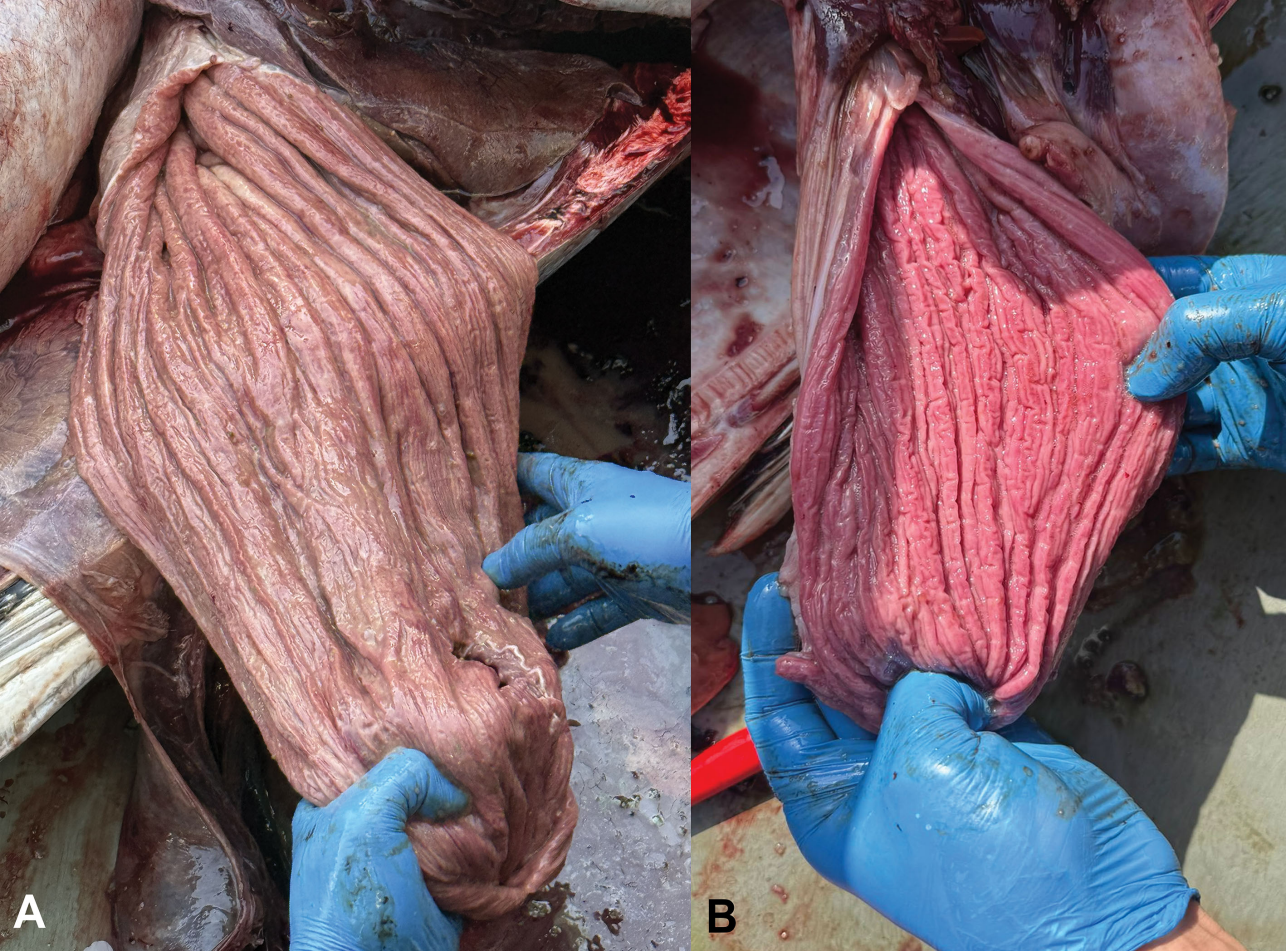
Gross gastric lesions observed during the 2025 mortality event in Atlantic bluefin tuna (*Thunnus thynnus*). **(A)** Mild diffuse reddening (hyperemia) of the gastric mucosa observed during early-stage disease. **(B)** Marked diffuse mucosal reddening and hemorrhagic appearance (“red stomach”) observed in mid-stage disease in moribund fish. Images are representative of affected individuals examined shortly after collection in Atlantic bluefin tuna (*Thunnus thynnus*).

Necropsy findings in smaller fish (mean 50 kg) included discrete pinpoint foci (petechia and/or mild hemorrhages) on the gastric and intestinal mucosa. In larger fish (mean 150 kg), initial examination revealed marked petechiae, ecchymoses, and larger hemorrhagic areas on the surface of the gonads and the parietal peritoneum, with patchy ecchymoses and moderate hyperemia and hemorrhage of the gastric and intestinal walls (“red stomach”) (**Figure 1**). Gross gastrointestinal mucosal color changes can be influenced by multiple factors; however, these findings were consistently observed in freshly collected and moribund fish examined shortly after capture. Therefore, gross descriptions are provided conservatively and interpreted in conjunction with histopathology and microbiological results.

**Figure 2:**
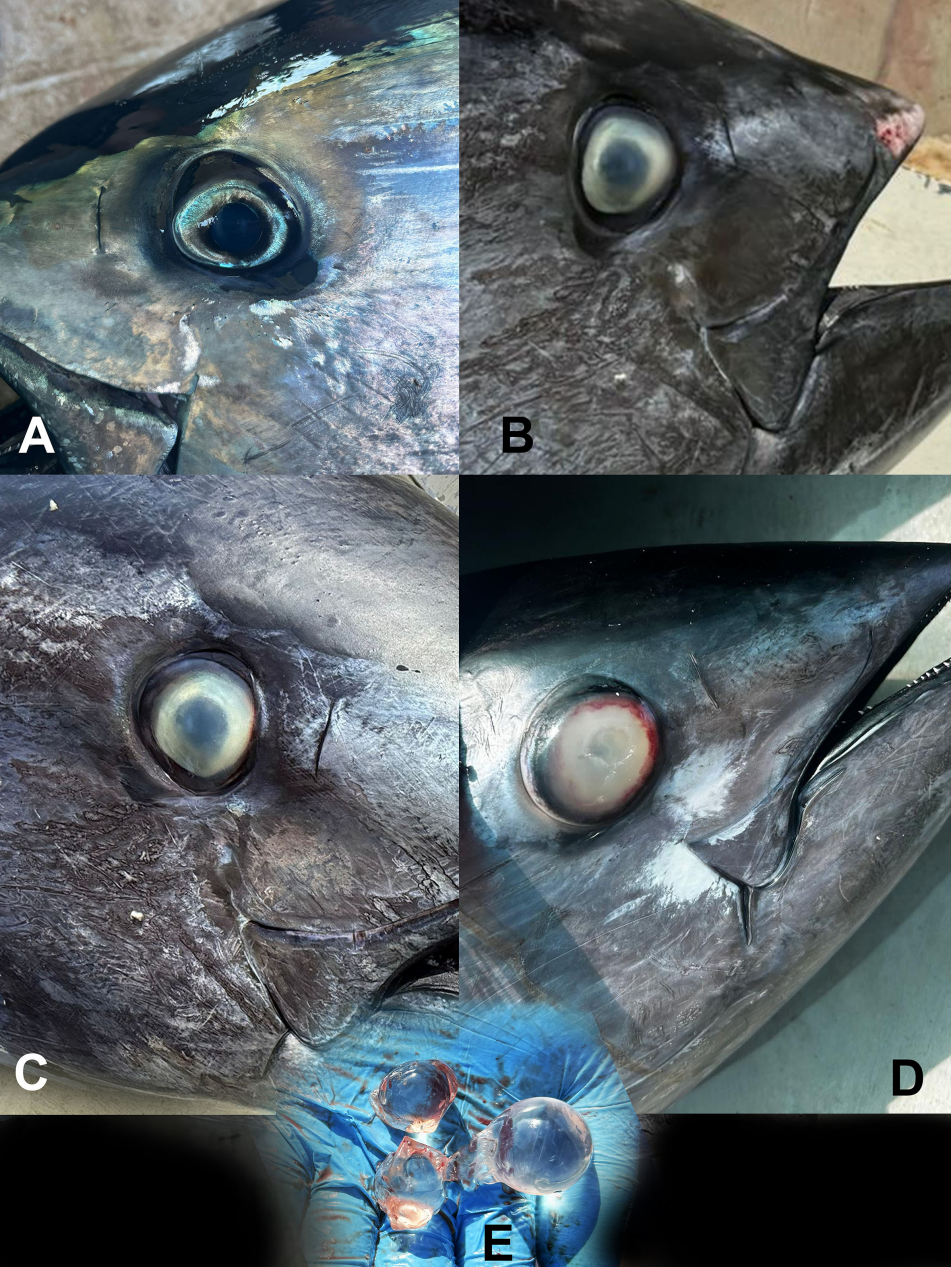
Gross ocular changes observed in affected Atlantic bluefin tuna (*Thunnus thynnus*) during the 2025 lactococcosis outbreak. **(A)** Normal eye from an apparently healthy individual. **(B–D)** Representative examples of progressive corneal opacity consistent with keratitis and/or corneal edema observed in moribund fish. **(E)** Example of lens appearance with apparent size variation.

**Figure 3:**
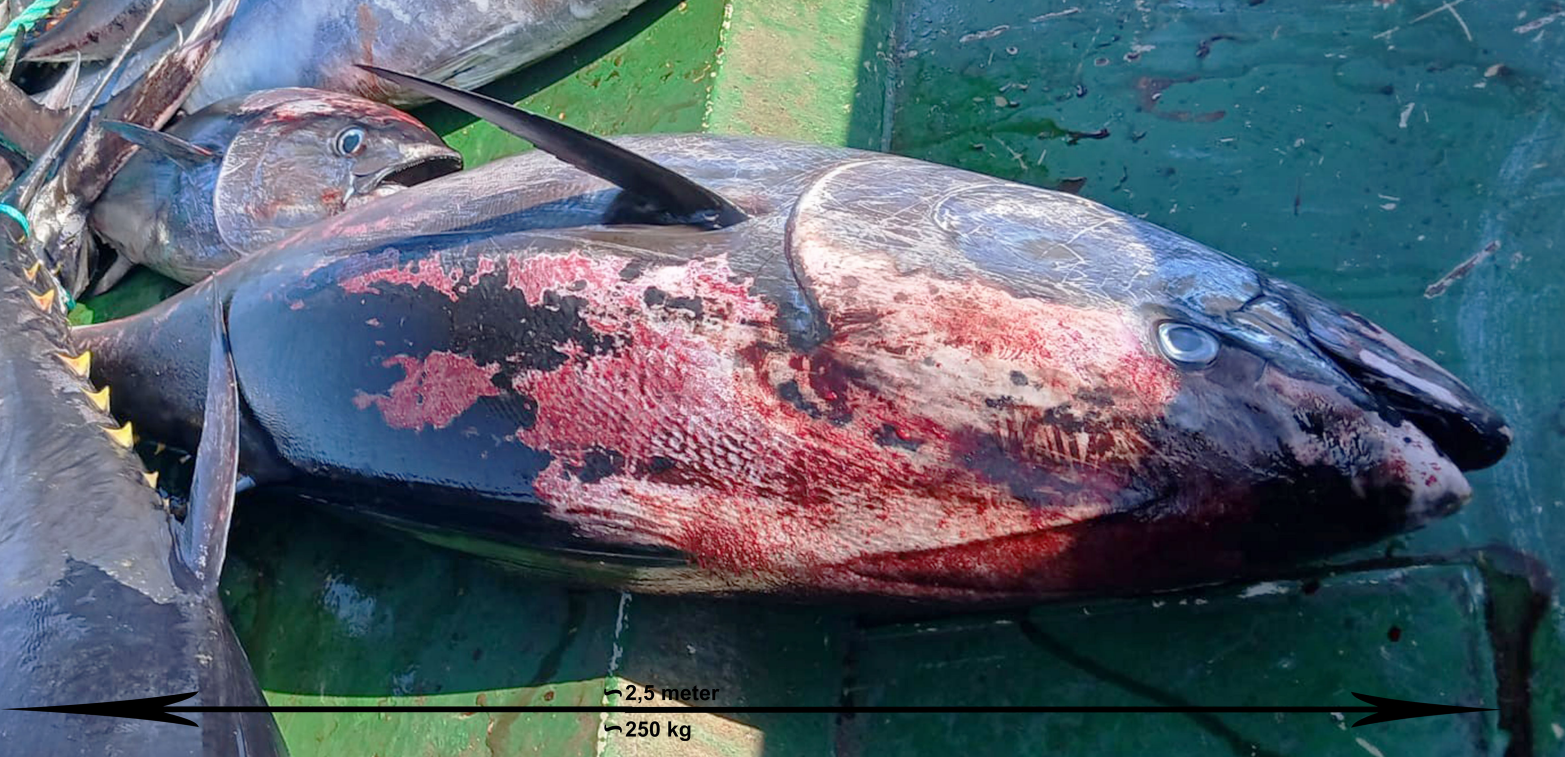
Severe cutaneous ulceration extending into the underlying muscle was observed in a subset of moribund fish in Atlantic bluefin tuna (*Thunnus thynnus*). The etiology and further characterization of cutaneous lesions cannot be definitively attributed without targeted histopathology; handling or autolysis is considered.

Approximately 10 days after the first signs, examinations of moribund smaller fish showed marked corneal opacity and suspected decreased vision (**Figure 2**), worsening gastrointestinal hemorrhage, necrotic foci in the heart, and hyperemic regions in the bulbus arteriosus. In larger fish (mean 150 kg), hemorrhagic and necrotic areas were present in the skeletal muscle, and necrotic foci were observed in the heart, with severe hemorrhage over the bulbus arteriosus. Reduced feed response and reduced awareness or avoidance were also observed. Decreased vision was considered a possible contributing factor but could not be confirmed without histopathological examination of the eye tissue (**Figure 3**). Lenses occasionally varied in diameter but remained translucent and blurred cornea was also observed in various severities.

**Figure 4:**
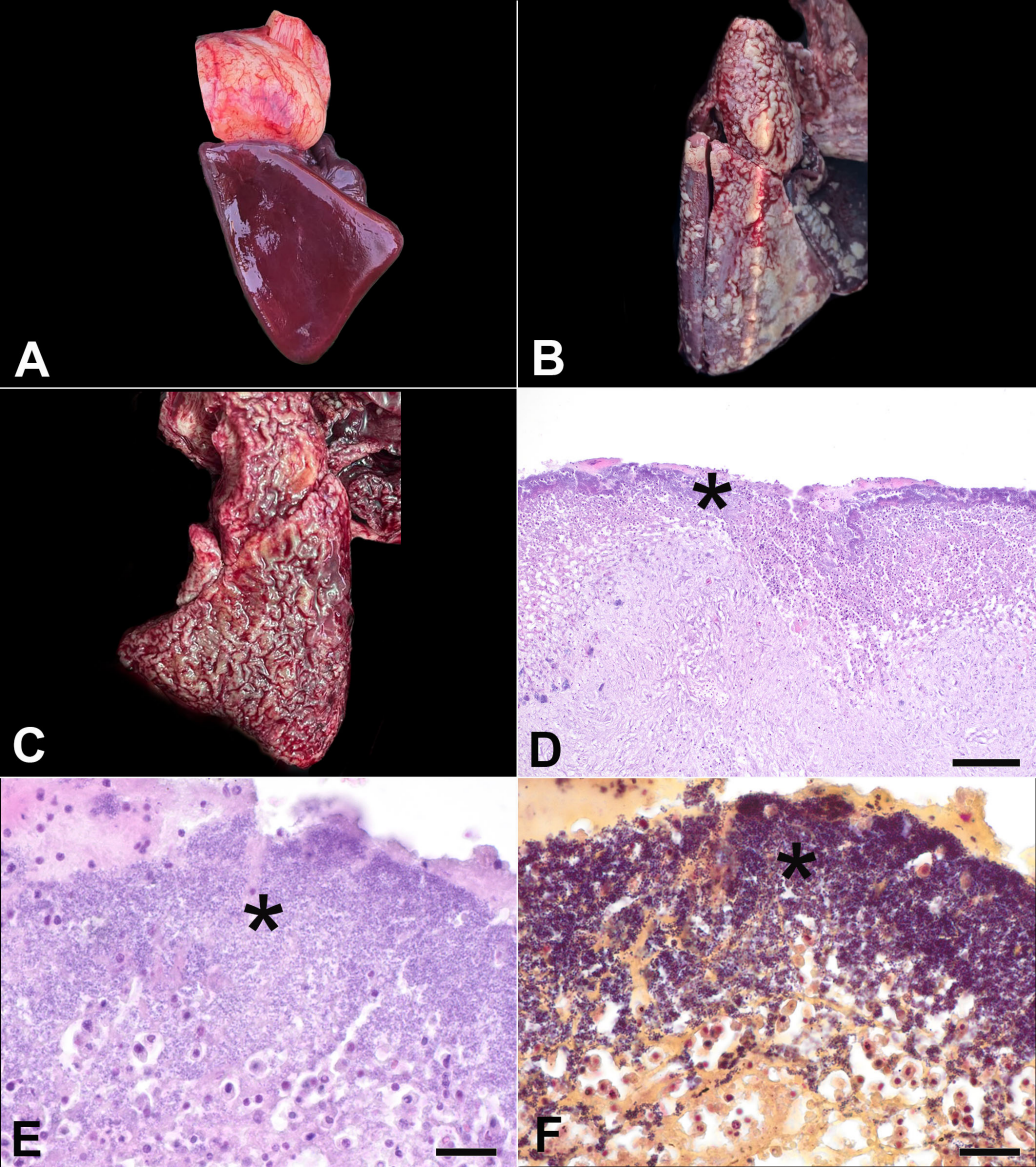
Gross appearance of the heart during the outbreak of Lactococcosis in tuna fish, **(A)** Seemingly healthy but mild hyperemia in the bulbus arteriosus section of the heart, **(B)** Severe fibrinous epicarditis affecting all heart compartments, **(C)** Severe fibrinous epicarditis affecting the atrium, ventricle, and bulbus arteriosus, **(D)** Photomicrograph of the ventricular epicardium revealing macrophages and cell debris overlain by a mat of fibrin and sheets of bacteria (asterisk), hematoxylin and eosin (HE) stain, Bar = 200 µm. **(E)** Photomicrograph of high magnification of the epicardial surface showing mats of intracellular and extracellular coccoid bacteria (asterisk) admixed with fibrin, cellular debris, and macrophages, HE stain, Bar = 20 µm, F: Photomicrograph of high magnification of a Gram stain of the same epicardial surface reveal large numbers of monomorphic Gram-positive intracellular and extracellular bacterial cocci (asterisk), Gram stain, Bar = 20 µm.

Ocular changes (corneal opacity and associated gross abnormalities) are commonly reported in lactococcosis caused by *L. garvieae* in multiple fish species and, in the present outbreak, occurred together with systemic lesions of septicemia and abundant intralesional Gram-positive cocci in affected organs (see **Figures 3**, **4**) and culture/16S evidence supporting *L. garvieae* as the dominant etiologic agent. The pathogenesis of the ocular lesions in Atlantic bluefin tuna requires further targeted investigation (e.g., dedicated ocular histopathology) and is highlighted as a priority for future studies.

By approximately 20 days after the onset of mortalities, external examination of sampled small and large moribund fish showed severe skin ulceration, extensive scale loss, subcutaneous hemorrhages, and marked hemorrhage within skeletal muscle (**Figure 3**), along with bilateral corneal opacities.

Internal examination of both smaller and larger fish demonstrated severe myocardial hemorrhage, a diffusely reddened bulbus arteriosus, and, on the epicardial surface of some hearts, prominent fibrin deposition (**Figures 4A–C**). Twenty days after the onset of mortalities, the liver was markedly friable and, on sectioning, exuded a tan fluid; the parietal peritoneum was diffusely covered by hemorrhage. Size variation in lenses was pronounced, and feeding trials suggested reduced vision with affected fish showing reduced awareness while swimming and being easily captured.

**Figure 5:**
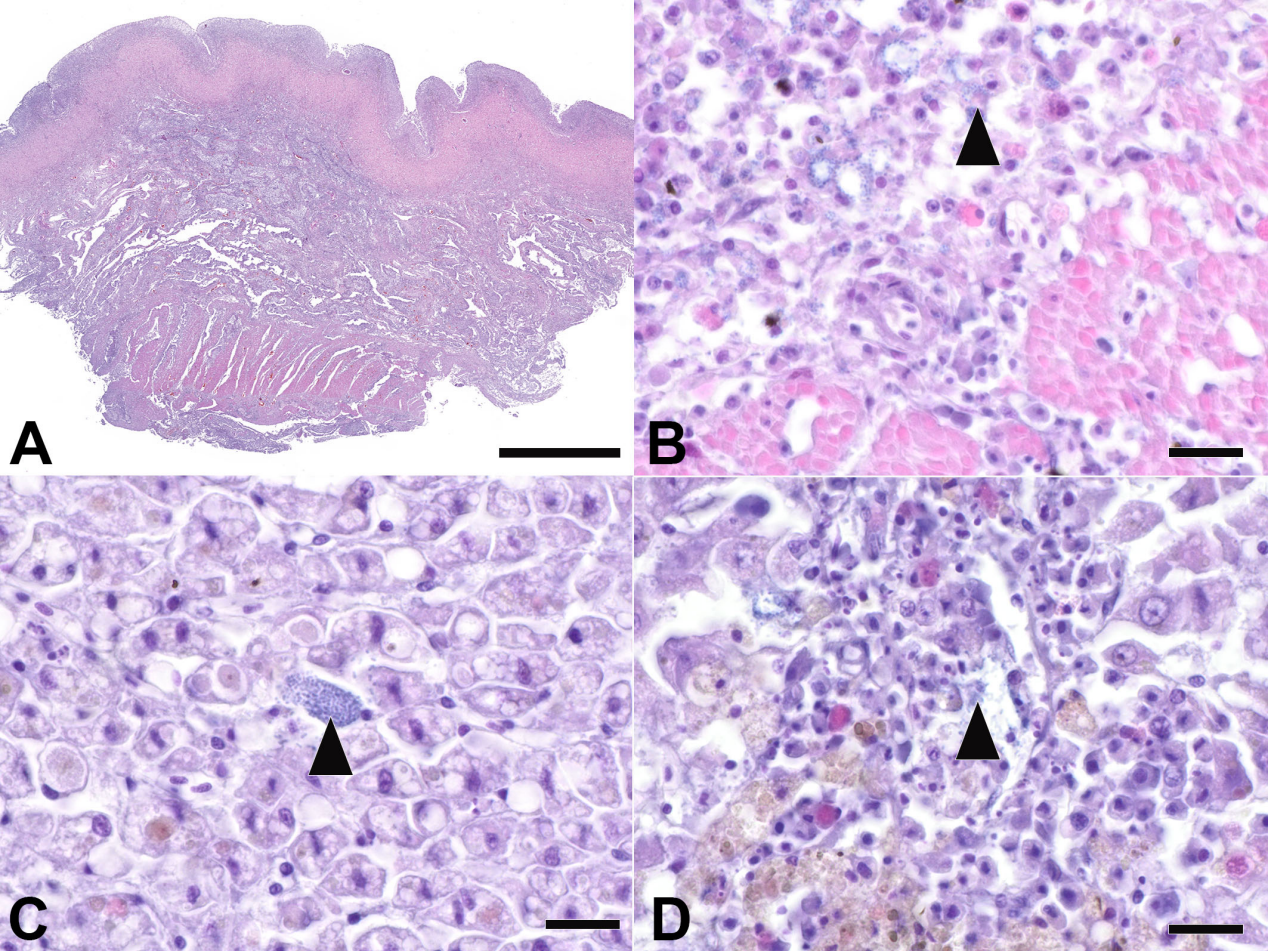
**(A)** Subgross histologic examination of the stomach reveals marked expansion of the submucosa, muscularis, and serosa by inflammation in Atlantic bluefin tuna (*Thunnus thynnus*), hematoxylin and eosin (HE) stain. Bar = 4 mm. **(B)** Higher magnification of the gastric muscularis shows numerous infiltrating macrophages, often containing intracytoplasmic coccoid bacteria (arrowhead), admixed with variable amounts of fibrin, cellular debris, and a few eosinophilic granular cells, HE stain. Bar = 20 µm. **(C)** Hepatic sinusoids often contain clusters of extracellular coccoid bacteria (arrowhead), HE stain. Bar = 20 µm. **(D)** Occasionally, clusters of extracellular coccoid bacteria (arrowhead) within the liver are associated with infiltration of macrophages, a few eosinophilic granular cells, and cellular debris, which effaced the surrounding hepatocytes, HE stain. Bar = 20 µm.

Consistent with the gross findings, histopathology examination revealed that the epicardium in all examined areas of the heart was expanded by dense sheets of macrophages, with few admixed lymphocytes and rare multinucleated giant cells (**Figures 4D–F**). Multifocally, large aggregates of fibrin form foci among the macrophages, often with numerous clusters of abundant intracellular and extracellular Gram-positive bacterial cocci along the periphery (**Figure 5**). Rare inflammatory cells infiltrate into the underlying myocardium and the interstitial spaces surrounding myocardial vessels.

The serosa of remaining tissues, including the spleen, ovary, and stomach, was variably expanded by similar aggregates of fibrin and inflammatory cells, occasionally with admixed coccoid bacteria (**Figures 5A, B**). Similar extracellular coccoid bacterial agents were scattered throughout the hepatic parenchyma within sinusoids (**Figure 5C**) and small clusters of mixed inflammatory cells (**Figure 5D**). Histologic findings, likely incidental or unrelated to the systemic bacterial disease, included presumed ovarian follicular atresia with oophoritis and rare granulomas suggestive of historic or chronic parasitism.

### 3.2 Bacterial growth, phenotypic characterization and primer identification

At the start of clinical signs, cultures from liver, spleen, and heart yielded uniform (pure) growth on TSA and BA. After subculture and confirmation of purity, colonies were white-pigmented, convex, circular, and smooth with entire margins. The isolates were Gram-positive cocci that occurred in pairs, triplets, or long chains; they were oxidase-negative, catalase-negative, non-motile, and fermentative in the oxidative/fermentative glucose test. Direct Gram stains of tissues showed organisms with the same morphology.

Biochemical assays demonstrated a consistent fermentative profile across isolates. Acid production was observed from glucose, ribose, mannitol, lactose, and trehalose, while sorbitol, raffinose, L-arabinose, and dulcitol were not utilized. Enzymatic reactions were positive for N-acetyl-glucosamine, pullulan, maltose, and melibiose, whereas Voges–Proskauer, alkaline phosphatase, and pyrrolidonyl arylamidase tests were consistently negative. This biochemical signature reflected a stable metabolic pattern, characterized by a broad carbohydrate fermentation capacity and selective enzymatic activity. MALDI-TOF MS analysis yielded a score of 2.2, indicating reliable species-level identification as *L. garvieae*. This result was corroborated by molecular assays: multiplex PCR produced the expected amplicon with the *L. garvieae*-specific LG_IBS primers (≈132 bp), whereas no amplification product was detected with the *L. petauri*-specific LP_IBS primers (583 bp).

### 3.3 16S amplicon sequencing results

**Figure 6:**
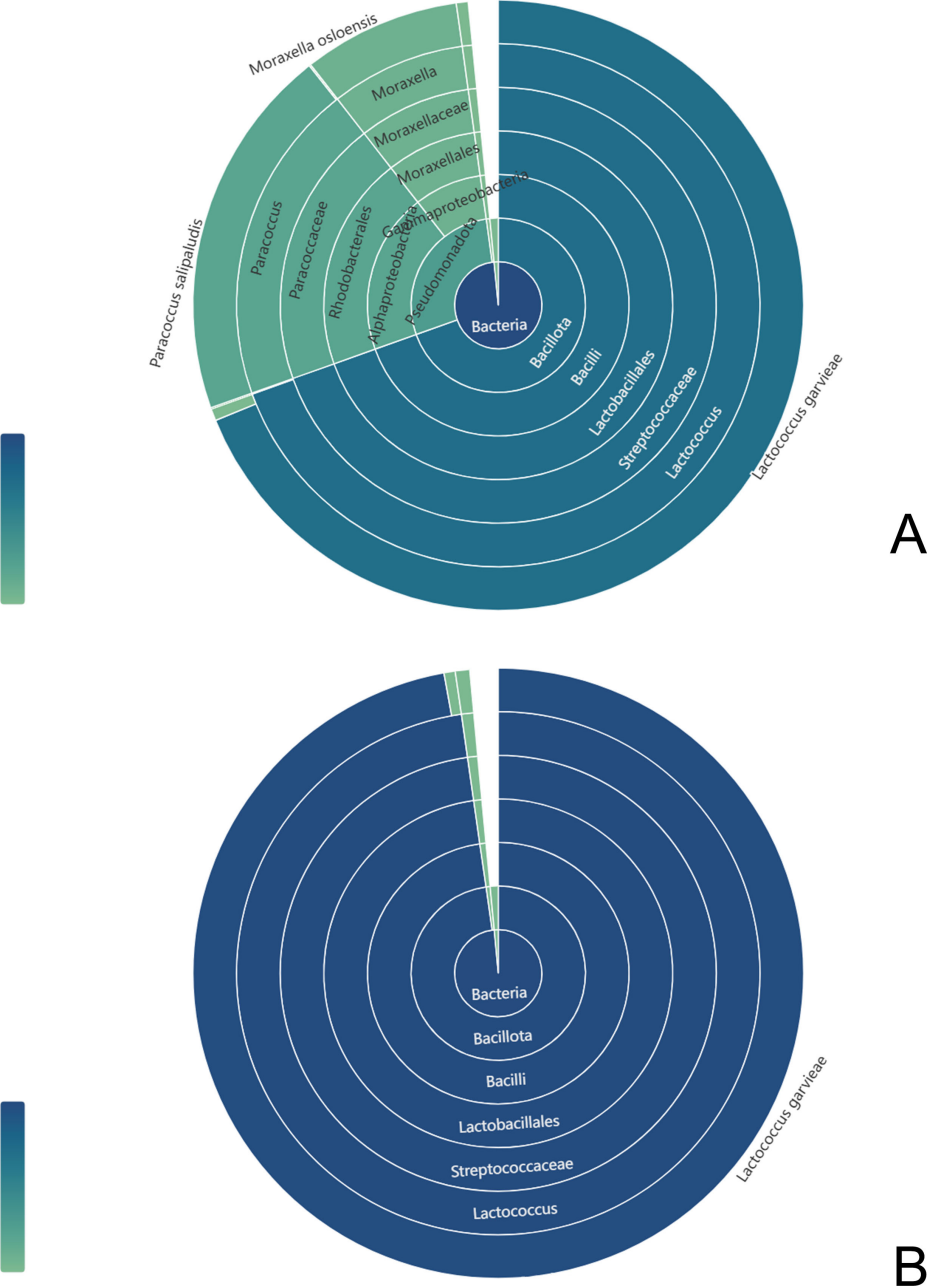
16S rRNA amplicon sequencing profiles of bacterial taxa in affected Atlantic bluefin tuna (*Thunnus thynnus*) tissues. **(A)** Spleen; **(B)** liver and heart. Bars show relative read abundance after full-length 16S (27F/1492R) Nanopore sequencing and taxonomic classification against the NCBI 16S/18S RefSeq database. Across tissues, *Lactococcus garvieae* dominated the profiles, supporting a primary systemic bacterial etiology.

Across all tissues, the dataset was overwhelmingly dominated by a single taxon, *L. garvieae* (3, 268/3, 706 reads; approximately 88.2%). At the per-sample level, *L. garvieae* constituted approximately 69.8% of reads in the affected spleen (875/1, 253), 97.2% in the liver (1, 746/1, 796), and 98.5% in the heart (647/657). Minor constituents were detected only in the spleen, including *Paracoccus salipaludis* (approximately 20.1%; 252 reads) and *Moraxella osloensis* (approximately 8.5%; 106 reads). The “Unknown” assignments accounted for a small fraction in each sample, including approximately 1.6% in the spleen, 2.8% in the liver, and 1.5% in the heart, suggesting limited unclassified signals. Taxonomically, the dominant organism belonged to Bacillota, class Bacilli, order Lactobacillales, family Streptococcaceae, and genus *Lactococcus*. The secondary taxa belonged to Pseudomonadota, specifically Alphaproteobacteria within Paracoccaceae/*Paracoccus* and Gammaproteobacteria within Moraxellaceae/*Moraxella*. Overall, this profile indicated a simple community structure rather than a diverse polymicrobial signal (**Figure 6**).

### 3.4 Complete genome analysis

**Figure 7:**
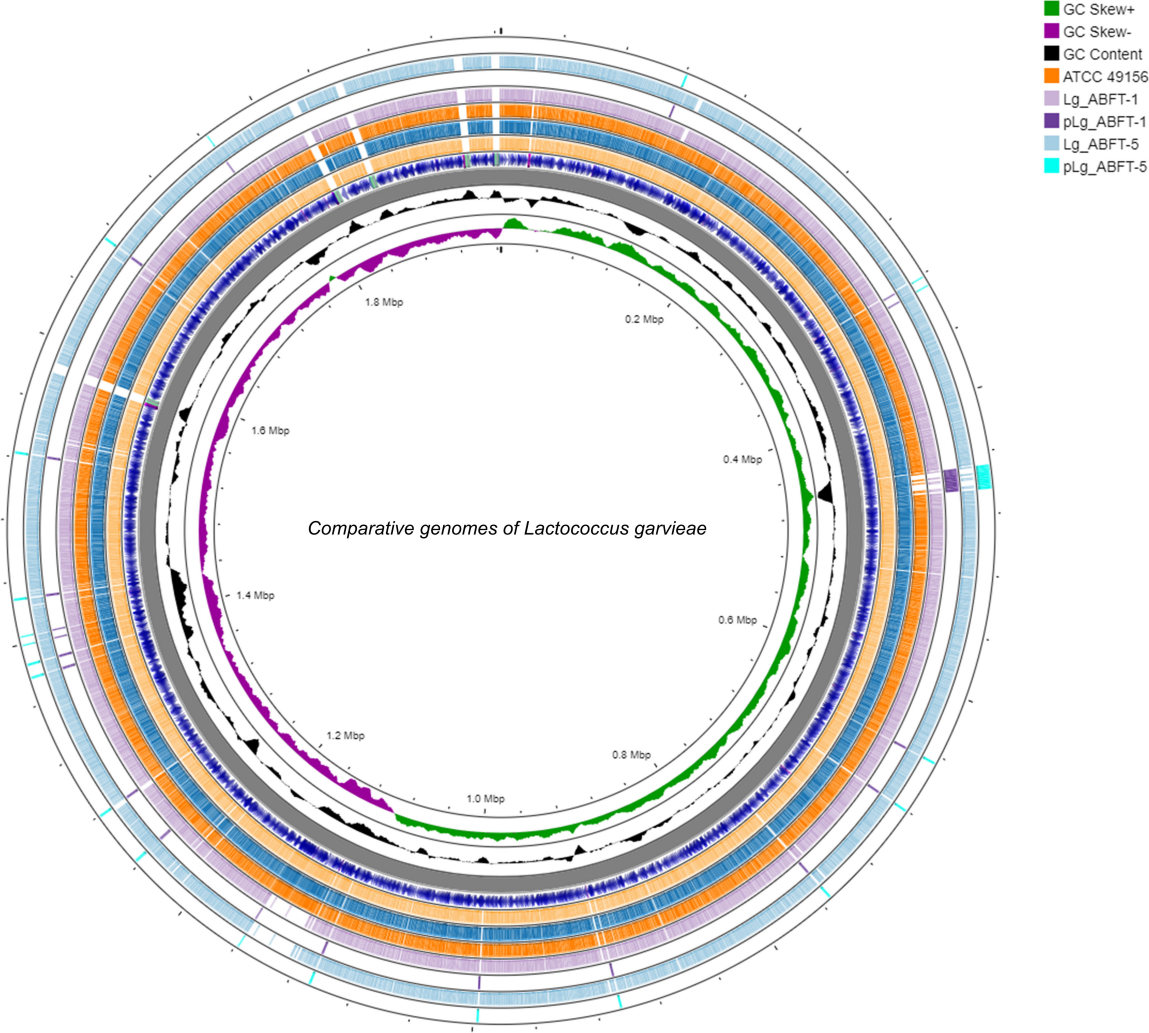
Comparison of the complete genomes of *Lactococcus garvieae* strains Lg2 (gray), JJJN1 (yellow), ATCC 49156 (orange), Lg_ABFT-1 (light blue), and its plasmid pLg_ABFT-1 (purple, located adjacent to Lg_ABFT-1), as well as Lg_ABFT-5 (sky blue) and its plasmid pLg_ABFT-5 (blue, located adjacent to Lg_ABFT-5). The inner rings represent GC content (black) and GC skew values, with positive skew (green) and negative skew (magenta), indicating potential replication origins and termini across the genomes.

Hybrid assemblies produced closed genomes for both isolates, each consisting of a single circular chromosome and a single circular plasmid. For Lg_ABFT-1, the chromosome measured 1, 945, 306 bp (GC 38.79%) and the plasmid pLg_ABFT-1 measured 31, 654 bp (GC 31.22%); the total genome length was 1, 976, 960 bp with an overall GC content of 38.67%, N50 equaled 1, 945, 306 bp and L50 was 1. For Lg_ABFT-5, the chromosome measured 1, 945, 316 bp (GC 38.79%) and the plasmid pLg_ABFT-5 measured 31, 654 bp (GC 31.22%); the total genome length was 1, 976, 970 bp with an overall GC content of 38.67%, N50 equaled 1, 945, 316 bp, and L50 was 1 (**Figure 7**). The complete chromosome and plasmid sequences of strains Lg_ABFT-1 and Lg_ABFT-5 were deposited in the NCBI GenBank database. The chromosome and plasmid accession numbers were CM142740 and JBUBQB010000002 for Lg_ABFT-1, and CM142739 and JBUBQC010000002 for Lg_ABFT-5, respectively.

**Figure 8:**
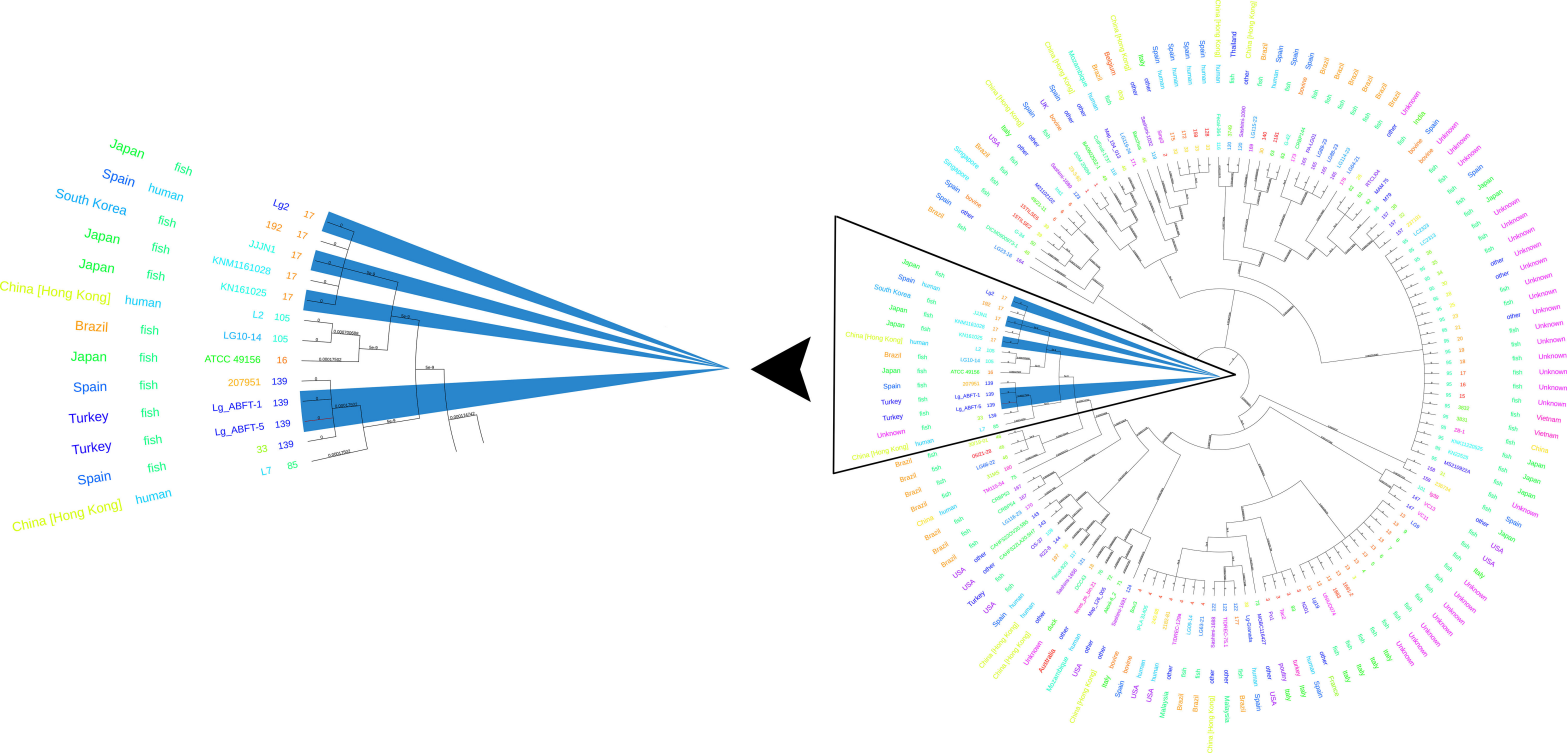
MLST-based phylogenetic tree of *Lactococcus garvieae* isolates. The tree was constructed from concatenated sequences of seven housekeeping genes (*als, atpA, galP, gapC, gyrB, rpoC, tuf*). Study isolates (Lg_ABFT-1 and Lg_ABFT-5) were assigned to ST139 and cluster with previously reported fish-associated isolates, including strains from Spain.

Digital DNA–DNA hybridization (dDDH) analysis performed using the Type Strain Genome Server (TYGS) confirmed the taxonomic placement of both isolates as *L. garvieae*. MLST analysis based on the seven-locus PubMLST scheme further assigned both study isolates (Lg_ABFT-1 and Lg_ABFT-5) to ST139. In the phylogenetic tree generated from concatenated MLST loci, the two isolates clustered closely with previously reported *L. garvieae* isolates from Spain, including isolates obtained from *Dicentrarchus labrax* (**Figure 8**).

To further resolve phylogenetic relationships among the analyzed genomes, a core-genome phylogeny was reconstructed from 98 taxonomically validated *L. garvieae* genomes. Panaroo analysis retained 1, 079 genes in the filtered core genome, resulting in a concatenated alignment of 1, 053, 534 nucleotide positions. Maximum-likelihood analysis placed the ABFT isolates (Lg_ABFT-1 and Lg_ABFT-5) within a clade comprising multiple fish-associated strains. Within this clade, the ABFT isolates were located near strains such as KN161025, JJJN1, and Lg2. Strains lacking capsule-associated features, including L21–12 and ATCC49156, were also distributed within the same broader phylogenetic cluster. Branch support values were high for several major nodes, while some internal nodes displayed lower support. The complete phylogenetic tree is presented in **Supplementary Figure 2**.

The whole-genome comparison shows that capsule-negative isolates lack the capsule locus at the position where it is present in other genomes. In strain Lg2, the capsule gene cluster is located on the chromosome, whereas in our isolates (Lg_ABFT-1 and Lg_ABFT-5), the capsule cluster resides on a separate plasmid. In ATCC 49156, the capsule cluster is absent; accordingly, the alignment over the corresponding region appears as an empty segment in this strain, yet its chromosome assembles as a continuous circular molecule without structural breaks, indicating true absence rather than an assembly gap. In Lg_ABFT-1 and Lg_ABFT-5, the chromosomes also assemble as complete circles, while the plasmid carrying the capsule locus is recovered as an additional circular replicon (**Figure 7**). In addition, the broader distribution of capsule-associated genes across the comparative *L. garvieae* dataset is summarized in **Supplementary Table 1**. A complete capsule operon profile was detected only in a limited number of strains, namely Lg_ABFT-1, Lg_ABFT-5, Lg2, JJJN1, and KN161025. By contrast, a small number of strains, including 3AA7, TM115-31, and TM115-54, exhibited only partial capsule-associated gene profiles, whereas the remaining genomes lacked capsule genes entirely. These findings indicate that the capsule operon is not broadly conserved within the species but is instead restricted to a limited subset of strains.

The complete plasmids carried by isolates Lg_ABFT-1 and Lg_ABFT-5 are small, low-GC replicons that each comprise ~30 predicted coding sequences distributed across ~32–33 kbp. Core plasmid maintenance functions (*rep*A/*rep*B replication genes and the partitioning gene *par*A) are present together with multiple mobile elements (IS982 copies and additional transposase/resolvase genes) and three short direct repeats (DR1–DR3). The capsule or exopolysaccharide (EPS) biosynthesis locus occupies the central part of the plasmid.

**Figure 9:**
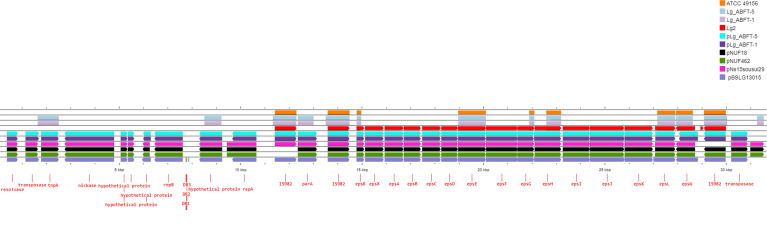
Linear comparison of the plasmid region of *Lactococcus garvieae*. Rows correspond to the following chromosomes or plasmids: ATCC 49156 (orange), Lg_ABFT-5 (light gray), Lg_ABFT-1 (lilac), Lg2 (red), pLg_ABFT-5 (cyan), pLg_ABFT-1 (purple), pNUF18 (black), pNUF462 (green), pNs15sousui29 (magenta), and pBSLG13015 (pale violet). Colored blocks denote homologous segments; their orientation reflects the direction of genes and synteny. The capsule gene cluster is about 16.5kb between IS982 and transposase.

Both plasmids encode a contiguous capsule and EPS cluster (e.g., *eps*R*, eps*X*, eps*A*–eps*D, a rhamnosyltransferase, additional glycosyltransferases, an acetyltransferase, *wzy* [repeat-unit polymerase], *wzx* [flippase], UDP-glucose 6-dehydrogenase, and the conserved *cps*L and *cps*W genes), bounded by IS982 elements. In the reference strain ATCC 49156, the entire ~16.5-kb capsule locus is absent, whereas the virulent strain Lg2 harbors an equivalent island (LCGL_0431–LCGL_0448) comprising the same functional set of genes; accordingly, relative to ATCC 49156 and other capsule-negative isolates, our isolates uniquely carry the capsule-associated genes listed above together with flanking IS982 transposase genes (**Figure 9**).

### 3.5 Antimicrobial resistance and virulence genes

Whole-genome analysis identified a single acquired resistance determinant shared by both isolates: *lsa*(D). This gene is responsible for encoding an ABC-F ribosomal protection protein, which is predicted to mediate target protection against lincosamides, streptogramin A, and pleuromutilins. No additional AMR genes were detected in either Lg_ABFT-1 or Lg_ABFT-5 (**Supplementary Table 2**).

Whole-genome screening showed that both of our isolates (Lg_ABFT-1, Lg_ABFT-5) harbor a broad set of virulence-associated genes: *hly-1/-2/-3*, NADH oxidase, *sod*, *pgm*, the adhesin genes a*dhPavA*, *adhPsaA*, *eno*, two LPxTG-anchored protein clusters (LPxTG-1–4), and the adhesin loci *adhCI* and *adhCII*. The generic adh locus was not detected. Critically, the complete capsule gene cluster was present in both isolates, indicating the genetic capacity for polysaccharide capsule biosynthesis—a trait linked to immune evasion and increased virulence in *Lactococcus*/*Lactobacillus* relatives. Among the surveyed determinants, the capsule locus was the most discriminative feature of our strains (**Supplementary Table 1**).

### 3.6 Transmission electron microscopy

**Figure 10:**
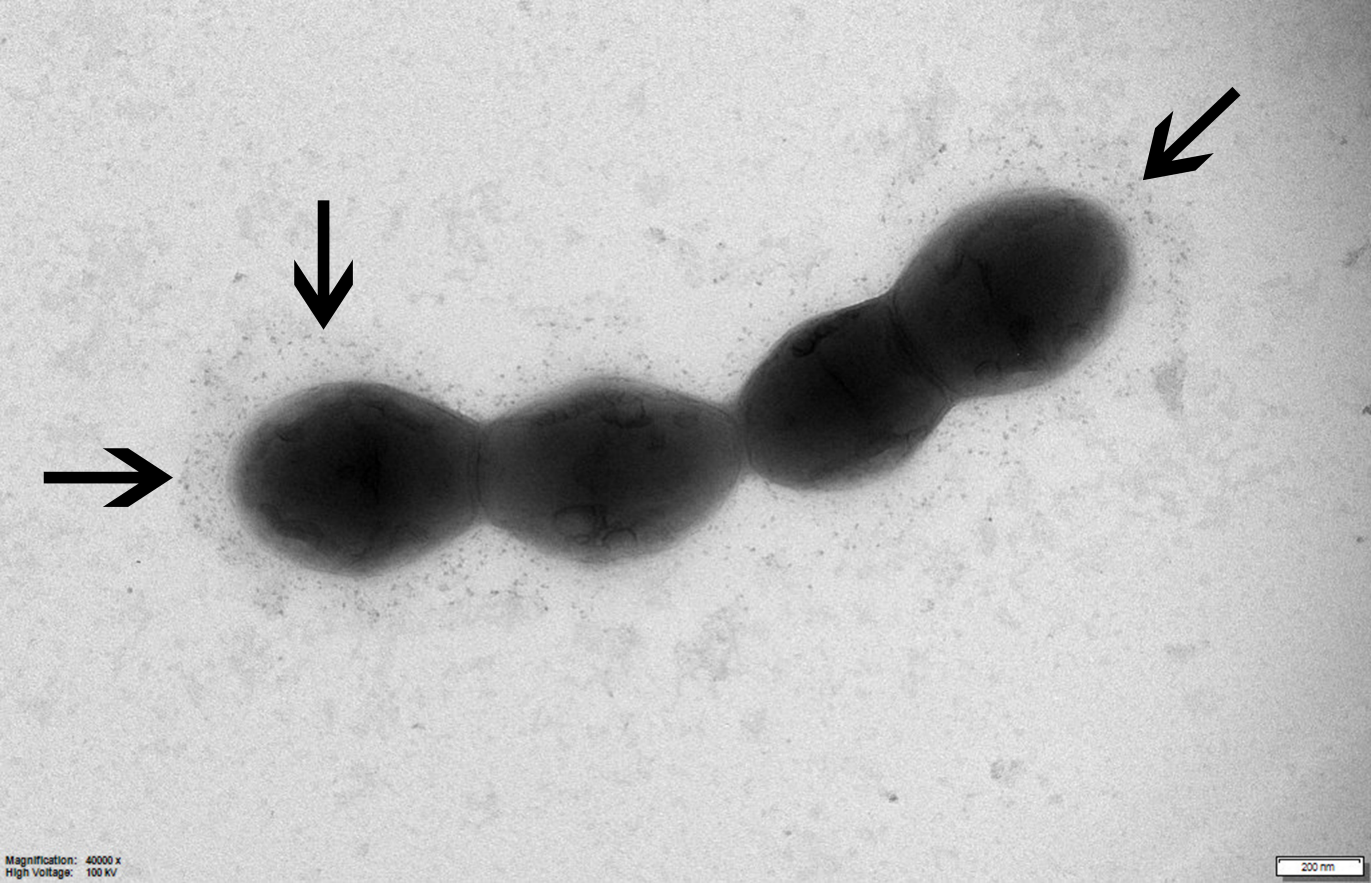
Capsule formation of *Lactococcus garvieae* strains (arrow shows well-developed capsule).

Approximately 100 nm cell capsules were observed in our *L. garvieae* isolates, even in the absence of immune serum treatment. According to the classification proposed by Ooyama et al. (2002), the capsule structure detected in this study was characterized as a well-developed cell capsule (**Figure 10**).

### 3.7 Antimicrobial susceptibility

Minimum inhibitory concentration (MIC) values and disc diffusion zones for florfenicol (FFC), oxytetracycline (OT), enrofloxacin (ENR), and erythromycin (ERM) obtained from the quality control (QC) strains were compared with the suggested ranges reported in CLSI VET03/VET04-S2 and fell within the specified limits. For erythromycin (ERM), a reference range is provided only for *Aeromonas salmonicida* subsp. *salmonicida*; accordingly, evaluation was performed using this QC strain, and the observed MICs fell within the specified limits. By contrast, because QC reference ranges are not provided for either QC strain for sulfamethoxazole (SUL) and doxycycline (DOX), QC-based suitability could not be assessed for these agents. For the study isolates, MIC breakpoints and disc zones for *L. garvieae* have not been established by CLSI or EUCAST; therefore, results were not categorized as susceptible or resistant and are reported as descriptive MIC data. For the study isolates, the MIC values were as follows: ERM: 0.016 (µg/ml); ENR: 0.064 (µg/ml); DOX: 0.064 (µg/ml); OT: 0.128 (µg/ml); FFC: 1 (µg/ml); SUL: 256 (µg/ml), and disc diffusion zones (mm) for the study isolate were ERM: 32; ENR: 22; DOX: 32; OT: 27; FFC: 31; SXT: 16 and CIP: 16.

## 4 Discussion

*Lactococcus garvieae* has been characterized as “an ever-present lethal threat” to fish species and to the regions where it has emerged, a risk amplified by the rising frequency of mortality events in marine environments under global warming (Meyburgh et al., 2017; Salogni et al., 2024). In recent years, during periods of elevated temperatures, it has caused high mortalities in species such as Nile tilapia (*Oreochromis niloticus*), rainbow trout (*Oncorhynchus mykiss*), yellowtail (*Seriola quinqueradiata*), amberjack (*Seriola dumerili*), cobia (*Rachycentron canadum*), barramundi (*Lates calcarifer*), and catfish (*Pseudoplatystoma* sp.), leading to substantial economic losses even when treatment is administered (Vendrell et al., 2006; Evans et al., 2009; Fukushima et al., 2017; Karami et al., 2019; Rao et al., 2022). However, to date, no disease attributable to *L. garvieae* and associated with high mortality has been reported in large tuna weighing 50–250 kg. Accordingly, this investigation constitutes the first report of a major outbreak causing mortality among the 1,500-2,00 stranded individuals, representing an estimated approximately 300,00 kg and more than $20 million in economic value. The concurrent observation of affected fish in nearshore and stranding contexts, and in the vicinity of cage-farming areas, underscores the need to integrate farm-level biosecurity or husbandry measures with nearshore monitoring and rapid diagnostics during warm-water periods.

In published reports of *L. garvieae* infections in other fish species, high levels of acute mortality predominate, with common clinical signs including anorexia, hyperpigmentation, lethargy, disorientation, and erratic swimming (Vendrell et al., 2006; Meyburgh et al., 2017; Duman et al., 2020). Commonly observed gross lesions include exophthalmia, corneal opacity, intraocular hemorrhage, cutaneous fin hemorrhages, swollen coelomic cavities, and widespread internal hemorrhages affecting the major viscera, such as spleen, liver, brain, gastrointestinal tract, kidney, and heart (Vendrell et al., 2006; Shahin et al., 2022; Egger et al., 2023). Microscopically, histologic lesions correlate with the described gross findings and represent Gram-positive coccoid bacterial sepsis consistent with lactococcosis. Described findings include disseminated inflammation in the eye, brain, kidney, and gastrointestinal tract, often with colonies of Gram-positive bacteria (Vendrell et al., 2006; Shahin et al., 2022). Overall, the clinical, gross, and histologic findings observed in Atlantic bluefin tuna (ABFT) in this study aligned with those previously described in other fish species infected with *L. garvieae*, particularly ocular opacity, gastrointestinal hemorrhage, fibrinous epicarditis, and intralesional Gram-positive cocci, as shown in **Figures 2**–**6**. Additional histologic study is warranted to further characterize and determine the significance of the ocular and cutaneous gross observations in ABFT. Findings in this study, not frequently described in other species, include variation in ocular lens size; the significance of this observation warrants additional investigation to determine its significance and underlying disease process, if any.

In contrast, few divergent lesion patterns have been reported in other fish species, whereas ABFT in this case series showed a stage-dependent spectrum of lesions over the course of the outbreak. During the first week after the onset of mortalities (predominantly smaller fish, ~50 kg), mortality was acute and was characterized by severe gastric and intestinal hemorrhage. In the limited gastric section examined histologically, inflammation and intralesional bacteria were most prominent in the serosa, muscularis, and submucosa, supporting serositis, hematogenous dissemination, or a combination of both rather than primary mucosal involvement. As the outbreak progressed into weeks 2–3 (increasingly involving larger fish, ~150 kg, although some smaller individuals also exhibited advanced lesions), lesions became more systemic, including intestinal hemorrhage, petechiae on the gonads, ecchymoses in the peritoneal cavity, and extensive hemorrhagic foci in the heart. In severe disease, marked fibrinous epicarditis, hepatic liquefaction, splenic abscessation, and corneal opacity without exophthalmos were recorded. In the latest stage, a cutaneous form characterized by deep ulcerations extending into the underlying musculature was also observed; this lesion type has not been previously reported in other fish hosts. Overall, these observations suggest that *L. garvieae*–associated disease in ABFT may progress from an acute gastrointestinal and serosal presentation to a systemic hemorrhagic and cardioperitoneal form, warranting further study given the limited comparable descriptions in the literature.

When compared with the biochemical characteristics of previously reported *L. garvieae* strains (differentiated from *L. petauri*) (Saticioglu et al., 2023), the phenotypic profile of the present isolates showed overall concordance with those previously described *L. garvieae* strains. The phenotypic profile of the present isolates was broadly consistent with previously described *L. garvieae* strains differentiated from *L. petauri*. This pattern corresponded closely to the reactions reported for *L. garvieae* ATCC 43921^T^ and OS-37 (Saticioglu et al., 2023). A minor difference was observed in mannitol fermentation: the present isolates were consistently positive, whereas *L. garvieae* strains in the study by (Saticioglu et al., 2023) displayed weak or variable reactions to this substrate. A minor difference was observed in mannitol fermentation, which was consistently positive in the present isolates, whereas *L. garvieae* strains in the study by Saticioglu et al. (2018) displayed weak or variable reactions for this substrate. This variation may reflect strain-specific metabolic differences or environmental adaptation related to the host species and geographic origin. In contrast, *L. petauri* isolates were characterized by positive reactions for sucrose, pectin, mucic acid, L-pyroglutamic acid, and inosine, all of which were negative in *L. garvieae* (Saticioglu et al., 2023). Among the overlapping traits examined, the most consistent discriminating feature between the two species remains sucrose utilization, which was positive in *L. petauri* and negative in *L. garvieae*. This distinction, together with the overall biochemical similarity to reference *L. garvieae* strains, supports the phenotypic identification of the present isolates as *L. garvieae*. Given the close relatedness among *Lactococcus* species implicated in lactococcosis, including *L. garvieae* and *L. petauri*, and the recognized potential for misidentification, species-level discrimination can be supported by the multiplex PCR assay developed by Saticioglu et al. (2023) using species-specific primer sets. In this context, Ustaoglu et al. (2024) reported apparent cross-amplification in their multiplex PCR assay involving the species-specific *gly* target for *L. garvieae* and the tag target for *L. petauri*, previously described by Saticioglu et al. (2023). Additional targets, namely the DUF1430 domain-containing protein (*duf*) for *L. garvieae* and the lichenan permease IIC component (*per*) for *L. petauri*, were also introduced for comparative molecular detection. However, in a recent study including strains isolated from both freshwater and marine fish, González-Martín et al. (2026) found no consistent cross-amplification among *gly* and *tag* target combinations. Notably, the *gly* target showed 100% specificity for *L. garvieae* and approximately two-fold greater detection sensitivity than *duf*, whereas the *tag* and *per* targets exhibited comparable diagnostic performance for *L. petauri*. These observations indicate that *gly* and *tag* represent robust and reliable molecular targets for the differentiation of *L. garvieae* and *L. petauri*, respectively.

From the sampled tissues, *L. garvieae* was isolated as the sole bacterial agent in culture, and 16S amplicon sequencing showed that *L. garvieae* dominated the liver and heart profiles, constituting more than 97% of reads in these tissues (**Figure 6**). In disease outbreaks in fish, few studies have employed 16S amplicon sequencing to determine the relative abundances of causative agents in tissues. While routine diagnosis typically relies on the identification of cultured bacteria using a range of microbiological techniques, this study combined routine microbiology with a metagenomic approach applied to lesioned tissues, enabling rapid and accurate etiologic diagnosis. This integrated workflow, comprising DNA extraction, PCR, and sequencing, enabled the identification of the disease agent within 24 hours with an accuracy exceeding 99%.

While multilocus sequence typing (MLST) analysis assigned the ABFT isolates to an established sequence type and indicated their relatedness to previously described fish-associated strains, the core-genome phylogeny provided a higher-resolution view of their evolutionary placement. The ABFT isolates clustered within a lineage containing several marine and aquaculture-associated *L. garvieae* strains, including KN161025, Lg2, and JJJN1. Notably, both capsulated and non-capsulated strains occurred within the same broader phylogenetic group, with capsulated isolates such as Lg2, KN161025, JJJN1, Lg_ABFT-1, and Lg_ABFT-5 positioned in proximity to non-capsulated strains, including L21–12 and ATCC 49156. These findings indicate that capsule carriage is not restricted to a deeply separated phylogenetic lineage, but instead occurs within closely related genomic backgrounds. Taken together, the MLST and core-genome analyses show that the ABFT isolates are embedded within the known diversity of *L. garvieae* rather than representing a distinct evolutionary lineage. Our isolates, Lg_ABFT-1 and Lg_ABFT-5, carried a complete capsule/EPS biosynthesis cluster on a small circular plasmid, whereas their chromosomes assembled as complete circular molecules without a chromosomal capsule island. This organization differs from that of the capsulated reference strain Lg2, in which the capsule locus is chromosomal, and from ATCC 49156, in which the capsule locus is absent (Ooyama et al., 2002; Kawanishi et al., 2006; Morita et al., 2011). Thus, the absence of a capsule island from the chromosomes of the ABFT isolates does not indicate gene loss or an assembly gap, but rather reflects the plasmid-borne location of the capsule cluster. The capsule plasmid contained genes involved in EPS regulation, glycosyltransferase activity, repeat-unit polymerization, flippase activity, and UDP-glucose metabolism, bounded by mobile genetic elements. This configuration suggests that capsule-associated genes may be horizontally mobile in some *L. garvieae* lineages, as also supported by previous reports describing variability in capsule-associated loci and plasmid-associated genomic regions among fish-pathogenic *L. garvieae* strains (Kawanishi et al., 2006; Kanai et al., 2017). Importantly, the genomic evidence was consistent with the TEM findings, which showed a well-developed capsule approximately 100 nm thick (**Figure 10**), in accordance with the capsule classification criteria described by Ooyama et al. (2002). Therefore, the ABFT isolates represent capsulated *L. garvieae* strains with a capsule-associated genomic configuration distinct from the chromosomal capsule locus described in Lg2.

According to Kawanishi et al. (2006) and Kanai et al. (2017), KG^-^ (capsulated) *L. garvieae* isolates have been obtained from a wide range of marine fish species cultured in Japan over a period of more than two decades, from 1992 to 2014. The earliest KG^-^ isolates were recovered from fish of the genus *Seriola*, including yellowtail (*Seriola quinqueradiata*), amberjack (*Seriola dumerili*), and kingfish (*Seriola lalandi*), collected from different prefectures between 1995 and 2002. In particular, strain KG9502 was isolated from yellowtail in 1995, while Lg2, Lg6, Lg8, Lg27, Lg38, Lg44, Lg74, Lg96, Lg122, and Lg147 were obtained from yellowtail, amberjack, or kingfish during 2002. All these KG^-^ isolates possessed a capsule and exhibited high virulence in yellowtail, causing 100% mortality in experimental infections (Kawanishi et al., 2006). Subsequent studies expanded the host range and temporal distribution of KG^-^ isolates: early isolates were again identified from yellowtail (*S. quinqueradiata*) in Nagasaki (BY-1, 1992; NUF752, 1995; NUF1051, 2007; NUF1070, 2008) and Kagoshima (KG9502, 1995), and from greater amberjack (*S. dumerili*) in Kagoshima (NUF1019, 2005). Additional KG^-^ isolates were detected in Japanese flounder (*Paralichthys olivaceus*) from Kagawa (KRS02016, 2002) and Nagasaki (Ns12sousui54, 2012), as well as in Pacific bluefin tuna (*Thunnus orientalis*) from Nagasaki (Ns12sousuiVH81, 2012). Notably, previous reports on the Nagasaki strain suggest that tuna have been infected with *L. garvieae* for approximately 13–15 years, and that captivity-related stressors together with recent rises in sea temperature driven by global climate warming have amplified the infection, culminating in devastating outbreaks. Following these reports, the present study represents the first detection of highly virulent, capsulated *L. garvieae* in the Mediterranean basin, isolated from ABFT. This finding demonstrates that the host range of *L. garvieae* has expanded beyond the species and geographic regions previously described, reaching new marine environments. Interestingly, recent surveillance studies conducted in the same region after 2020 found no capsule gene cluster in any of the *L. garvieae* isolates (n = 40) obtained from rainbow trout (*Oncorhynchus mykiss*), suggesting that capsulated strains are currently absent or rare in freshwater aquaculture systems (Altun et al., 2025). Notably, this host expansion occurred 11 years after the last reported KG^-^ isolates (2014) and resulted in severe disease outbreaks in Atlantic bluefin tuna, characterized by very high mortality rates comparable to those described in 2002, 2007, and 2014. The outbreak is estimated to have caused the largest economic loss attributed to *L. garvieae* infections to date, exceeding €10 million in the Mediterranean aquaculture sector for seabass and seabream (expected loss) and approximately €10 million for tuna in 2025.

Whole-genome screening showed that both of our isolates (Lg_ABFT-1, Lg_ABFT-5) harbor a broad set of virulence-associated genes, adhesin genes and the adhesin loci. The generic adh locus was not detected. Notably, the complete capsule gene cluster was present in both isolates, indicating the genetic capacity for polysaccharide capsule biosynthesis —a trait linked to immune evasion and increased virulence in *Lactococcus*/*Lactobacillus* relatives. Among the surveyed determinants, the capsule locus was the most discriminative feature of our strains. Across the comparative dataset, most virulence genes detected in our isolates (hemolysins, oxidative-stress enzymes, adhesins, LPxTG proteins) were widely distributed. In contrast, the capsule cluster occurred only in a minority of genomes, limited to a few strains (such as JJJN1, KN161025, Lg2) in addition to Lg_ABFT-1 and Lg_ABFT-5, and was absent from the majority of food or environmental isolates (such as LG728, LG791). This pattern suggests that the capsule locus is not ubiquitous in the species and may mark lineages with greater invasive potential (Morita et al., 2011; Miyauchi et al., 2012). Given its prominence in our genomes, targeted phenotypic confirmation (capsule staining or quantification, serum survival, or opsonophagocytic killing assays) would be valuable for verifying expression and assessing its contribution to virulence in our isolates. Together with these virulence-associated features, the therapeutic and stewardship implications depend on the resistance profile, which we interpret below using available genotype–phenotype data and published epidemiological cut-offs.

Whole-genome analysis identified only the ABC-F gene *lsa*(D), consistent with predicted ribosomal target protection against lincosamides/pleuromutilins rather than macrolides. This narrow resistome aligns with the observed phenotype (low minimum inhibitory concentrations (MICs) for ERM, OT, DOX, ENR, and FFC, and large inhibition zones for ERM/OT/FFC), indicating strong genotype–phenotype concordance overall. Using the proposed epidemiological cut-off values (ECVNRI) for *L. garvieae* (Öztürk et al., 2024), MICs for ERM, OT, and FFC were below wild-type thresholds (ERM <4 µg/mL, OT <2 µg/mL, FFC <16 µg/mL); the SUL MIC (256 µg/mL) was also below the reported SXT ECV (512 µg/mL) but should be interpreted cautiously due to agent mismatch. While ENR and DOX are not covered by the ECV tables in Öztürk et al. (2024), their low MICs suggest likely susceptibility; additional ECV information from other sources has been reported for some agents (Chan et al., 2025). Consistent with MIC-based interpretation, disk diffusion ECVs (Rosário et al., 2024) classified the isolate as WT for ERM (32 mm), FFC (31 mm), and OT (27 mm), whereas ECVs were unavailable for SXT, CIP, ENR, and DOX. The substantial inhibition zones observed for enrofloxacin and doxycycline suggest that our isolates are likely wild-type susceptible to these agents and may therefore respond favorably to therapy.

## 5 Conclusion

This study documents a severe mortality event in Atlantic bluefin tuna during summer 2025 and provides convergent evidence that *L. garvieae* was the primary etiologic agent, supported by culture-based identification, targeted molecular confirmation, tissue 16S amplicon profiling, and closed whole-genome sequencing. The isolates exhibited a capsulated phenotype, as confirmed by transmission electron microscopy. In addition, both isolates carried a capsule-associated biosynthesis locus, a feature that may contribute to enhanced invasive potential. Pathological findings were consistent with fulminant septicemia and prominent cardioperitoneal involvement, including fibrinous epicarditis with abundant Gram-positive cocci. Collectively, these findings expand the host and geographic context of capsulated *L. garvieae*–associated disease and highlight the importance of rapid etiologic confirmation and targeted prevention strategies during warm-water periods.

*L. garvieae* has been isolated in a variety of serotypes and across multiple fish species, resulting in significant economic losses. The most recent figures indicate that, in 2025, losses reached €12.5 million for gilthead seabream and European seabass, whereas the present study found an impact of over € 10 million on ABFT. State-of-the-art genomic characterization has identified the causative agent as capsulated *L. garvieae*, a variant not reported since 2002. These observations demonstrate that each lactococcosis outbreak may differ from previously documented cases, underscoring the critical importance of rigorous pathogen characterization to inform treatment and control programs. With chronicity, the infection can potentially progress from mild hemorrhage to severe lesions of the musculature, heart, gastrointestinal tract, eyes, and subsequently the skin within as little as 15 days. This highlights the importance of early therapeutic intervention and warrants additional study on disease progression and pathogenesis in these high-value fish.

## 6 Suggestions

● In conventional cage systems, fish welfare is primarily influenced by key parameters, including stocking density, water flow, oxygen availability, and thermal stability. Optimal welfare-oriented stocking densities typically fall within the range of 2–6 kg/m³, with initial phases often starting at 2–3 kg/m³, and some newer operations aiming for values of up to 10 kg/m³. Based on a cylindrical cage structure with an estimated radius of approximately 27.5 meters, the volume of the lower 15-meter section, where fish are primarily reared under thermally stable and welfare-compliant conditions, is calculated to be approximately 11, 874 m³, while the upper 25-meter section has an additional volume of approximately 59, 395 m³. Assuming a conventional cage structure with a usable vertical living space limited to the bottom 15 meters, and considering an average individual fish weight of 100 kg as typically observed in tuna farming, the estimated capacity under welfare-compliant conditions would accommodate approximately 350 individuals. This estimation assumes that only the lower part of the cage is suitable for continuous habitation, given the thermal stress observed in surface layers, particularly when sea temperatures exceed 25–26 °C.

**Figure 11:**
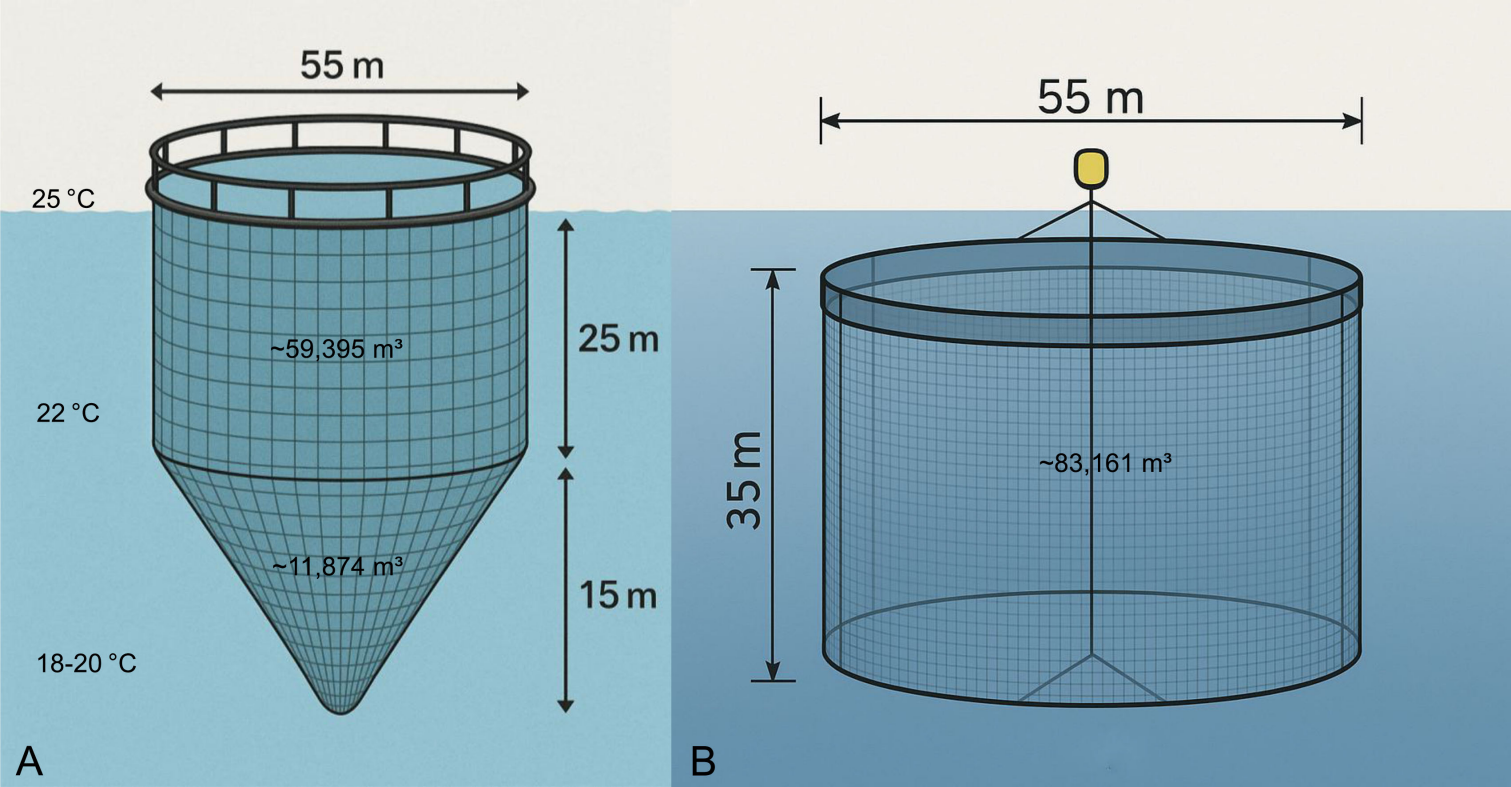
Conventional and suggested sea-cage configurations for Atlantic bluefin tuna (*Thunnus thynnus*) farming, shown with representative seawater temperatures for August 2025 in the Aegean Sea. **(A)** Conventional cage type. **(B)** Suggested cage system designed to promote more uniform vertical distribution of fish and improved use of the water column during warm-water periods. Temperature values are presented as approximate/estimated profiles for the period.

In the suggested cage system, which enables uniform vertical distribution of fish throughout the entire structure, the full 35-meter depth is utilized efficiently without restricting fish to thermally stable lower layers. Assuming a cylindrical geometry with an estimated radius of 27.5 meters, the total usable volume of the cage is approximately 83, 161 m³, representing a significant increase in habitable space compared to conventional systems limited to partial depth utilization (**Figure 11**).

*Management implications (practical recommendations for producers)*.

- Where feasible, install shade canopies to reduce solar heat gain or UV exposure and limit surface residency during hot periods.
- Prefer higher-flushing sites; align cages with prevailing currents to improve mixing, oxygenation, and heat dissipation in warm water.
- Shift feeding to dawn, dusk and deliver feed deeper in the cage column to reduce surface crowding when the temperature and irradiance peaks.
- When seawater approaches ≥24 °C, reduce ration size (and avoid heavy meals); consider periodic fasting during prolonged heat to lower metabolic load and waste output.
- Under non-peak thermal conditions, maintain a consistent (e.g., twice-daily) feeding schedule to support growth and avoid large single-meal crowding and oxygen spikes.
- At the first signs of abnormal behavior, lesions, or unexplained mortality, initiate immediate diagnostic sampling (bacteriology ± parasitology and histopathology) and document cases for rapid intervention.

If antimicrobial therapy is indicated, implement it under veterinary supervision with a standardized, auditable medicated-bait delivery protocol to ensure accurate dosing, traceability, and withdrawal-period compliance. 

## Data Availability

The datasets presented in this study can be found in online repositories. The names of the repository/repositories and accession number(s) can be found in the article/**Supplementary Material**.
