# Supplementary figures and images for: Capsulated Lactococcus garvieae caused devastating mortality in Atlantic bluefin tuna, Thunnus thynnus: genomic and histopathologic characterization
Source: Front Cell Infect Microbiol. 2026 Jul 3;16:1831351. doi: 10.3389/fcimb.2026.1831351 (PMC13375883; doi:10.3389/fcimb.2026.1831351)

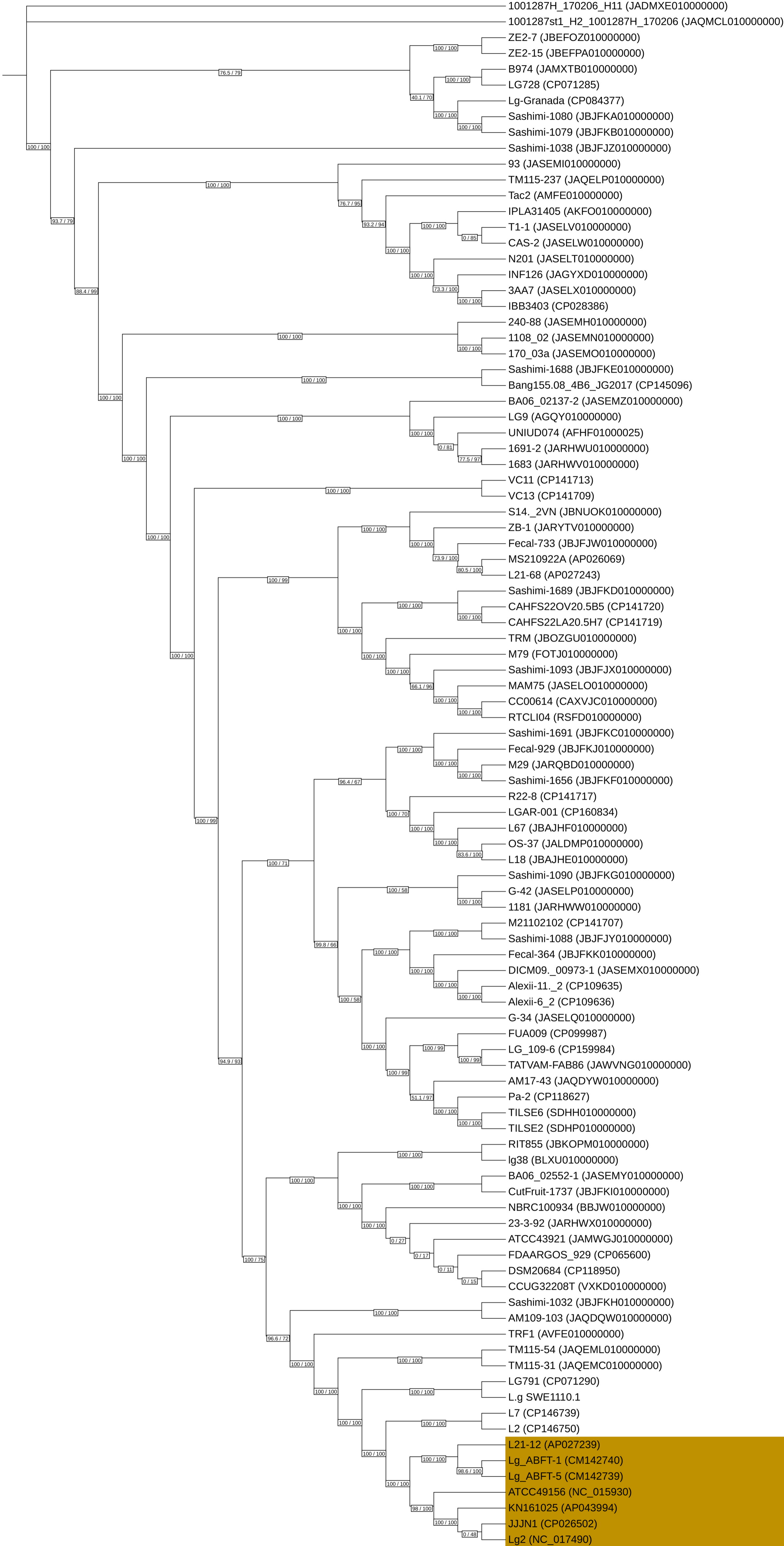

Supplement: Supplementary file 4 [file Image2.pdf]
